# Setting Up a Medical Oncology Educational Program in Sub-Saharan Africa

**DOI:** 10.5334/aogh.2994

**Published:** 2021-08-12

**Authors:** Sara Bravaccini, Patrizia Serra, Dino Amadori, Mattia Altini, Nestory Masalu, Deogratias Katabalo, Lauro Bucchi, Carla Masini

**Affiliations:** 1 IRCCS Istituto Romagnolo per lo Studio dei Tumori (IRST) “Dino Amadori”, Meldola, Italy; 2 Bugando Medical Center, Mwanza, Tanzania

## Abstract

One of the major problems facing healthcare systems in countries with poor socio-economic conditions is the need to strengthen the system through the training of physicians, nurses and other healthcare operators. Partnering with more affluent countries is the key for hospitals in these areas, but such alliances are often based on limited educational exchanges. We present a retrospective study of our experience in building a collaborative relationship between our cancer institute in Italy and a Medical Center in sub-Saharan Africa (Tanzania). The main purpose is to see the changes in the clinical practice after educational interventions on health personnel in a Tanzanian cancer center.

## Introduction

In Africa, the current estimated annual number of new cancer cases is 1,109,209, for a crude incidence rate of 82.7 per 100,000 persons and an age-standardised (world) rate of 132 [1]. Particularly in sub-Saharan Africa [2,3,4], comprehensive prevention, detection, and treatment programmes for cancer are lacking, the health infrastructure is poor, the technical workforce is limited, and diagnostic and treatment capacity is insufficient [2]. In Tanzania, where more than 35,000 new cancer cases are diagnosed each year; the case-fatality ratio approaches 80% [5]. There is a lack of awareness of cancer as a serious illness with some people still believing that it is an infectious disease. The lack of available diagnostic resources lead to late detection and late diagnosis and, therefore, to more advanced stages of disease. This, coupled with inadequate treatment, causes survival rates to be very low. Cancer follow-ups are also insufficient because constrained economic conditions make it difficult for patients to reach hospitals. Given that the workforce, infrastructures, and technologies are underdeveloped and their upgrades are difficult, public health campaigns and quality assurance are more important than in other more-developed countries [6,7]. Within this context, the lack of adequate personnel training is even more critical. Scientific organizations and cancer centers of the developed world could make a positive impact by becoming actively involved in organizing health educational programmes, international collaborative studies, and training courses undertaken locally and abroad. Given that offering scientific/medical training and access to research programmes could help to increase the capacity of local healthcare professionals, several Italian educational projects have been activated in recent years to promote such initiatives.

The poor awareness about cancer in both the general population and healthcare personnel prompted us to create a project that would provide medical and nursing staff with essential information to facilitate the the management of the disease. In view of the fact that few literature data are available on educational programs in oncology in sub-Saharan Africa, we felt that it would be important to report our experience of the impact of our educational program on the staff of hospital based in a low-income country. We performed a retrospective study to verify the changes that occurred in clinical practice following educational interventions in a Tanzanian cancer center.

## Methods

In 2000, a cancer institute and a cancer volunteer association (Associazione Vittorio Tison) based in northeast Italy, working in collaboration with Tanzanian political and health authorities, opened a Pathology Laboratory (considered to be a core element of a cancer care and control programme) and subsequently a Medical Oncology Unit at the Bugando Medical Center (BMC) in Mwanza, the largest referral hospital in the Lake zone of North-West Tanzania [8,9]. The partnership led to the creation of the Mwanza Cancer Project, sponsored by both Italian organizations [8].

From an educational point of view, the main intervention took the form of a five-year period of training in oncology (2004–2008) in Italy offered to a Tanzanian physician and was completely funded by Associazione Vittorio Tison. Thanks to their economic support, the physician was able to gain valuable, practical experience in the routine activities of the Department of Medical Oncology of our institute (IRST IRCCS), whilst also studying for a Specialization in Medical Oncology at Ferrara University. Thanks to a Memorandum of Understanding established in 2008 between BMC and the Italian partnership, an Oncology Day Hospital and Inpatient Ward were subsequently opened at BMC in Mwanza under the responsibility of the same oncologist.

IRST provides drugs, normally paid for by patients, free of charge to BMC. A team of rotating volunteers from IRST, comprising of physicians, pharmacists, nurses, data managers, began to visit BMC. They brought their own expertise to the courses organized on medical oncology, biology, technical aspects and correct preparation of cytotoxic agents, and data management for clinical trials. The visiting teams went to the hospital every 3–4 months, as agreed by Dr. Nestory Masalu, Director of the BMC Oncology Unit. Together, they decided on how to best structure the time spent at the center. The visitors also documented their stay by collecting data and information on the impact of the medical education program.

**Figure 1:**
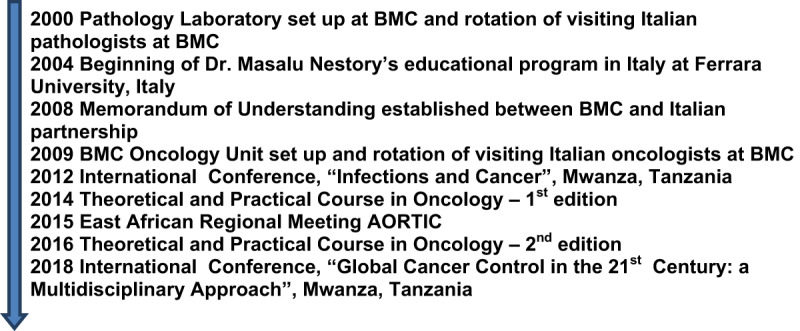
Timeline of the educational program.

The principal milestones of the education program are reported in ***Figure 1***.

## Results

**Figure 2:**
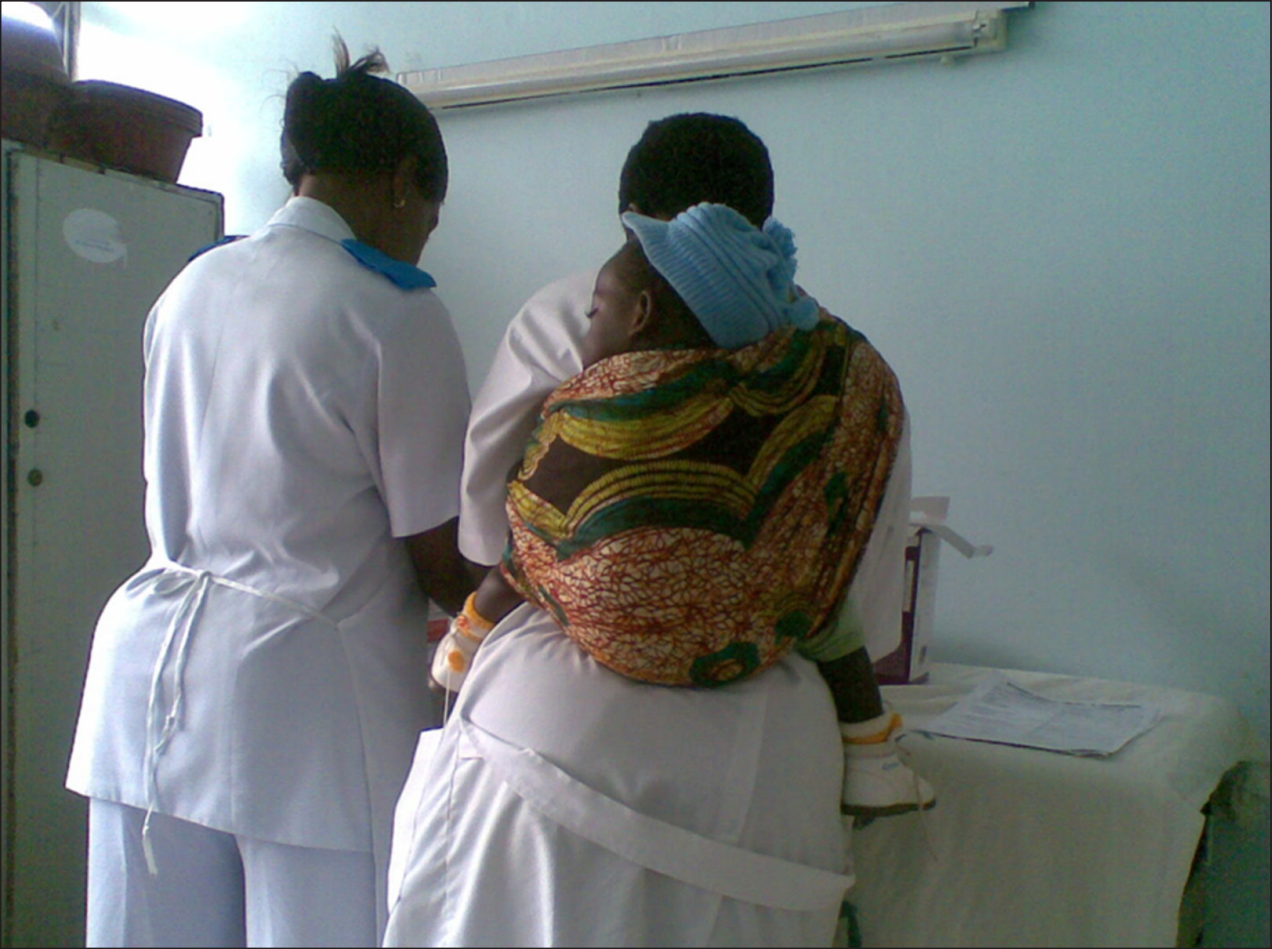
Tanzanian nurses preparing chemotherapy (February 2010).

Given the level of our clinical healthcare and automated procedures in terms of technical know-how, the impact of our personnel on the quality of medical care at BMC has been substantial. At the beginning of the project, Italian medical volunteers visiting Mwanza were surprised to see that patients tended to choose their beds at random each night and that more than one patient often shared the same bed. This obviously increased the risk of error in drug administration. Moreover, beds were sometimes used as a table for nurses to prepare chemotherapy. Like hospitals in Italy 30 years ago, chemotherapy at BMC was prepared by unskilled personnel who did not have the protection of a laminar flow hood. On one occasion at BMC, a volunteer recalls seeing two nurses, one with her baby on her back, preparing chemotherapy in the Hospital Nursery which, fortunately, was closed at the time (***Figure 2***).

At BMC, the quality of cancer treatments has improved considerably since visiting IRST pharmacy and the nursing personnel began teaching Tanzanian hospital workers about medical procedures and activities related to cancer drugs. They have also provided BMC staff with essential information on several important issues, such as handling procedures for the preparation of cytotoxic agents to guarantee the safety of the operator and the environment, need for prescription accuracy (which dose and which drugs) for specific types of cancer, importance of maintaining sterility during the entire process. Other key aspects of drug preparation have also been focused on cleaning and disinfecting of the handling area, the use of protective equipment, and proper hand washing (***Table 1***).

**Table 1:** Impact of medical education program on Bugando Medical Centre.


Before medical education program (up to 2015)	After medical education program (after 2015)

Improper use of beds (also sometimes used to prepare chemotherapy).	Beds only used for sleeping.

Patients chose beds at random each night and beds often shared by more than one patient.	Same beds used for one patient only.

Photosensitive drugs not covered.	Photosensitive drugs covered.

Incorrect drug administration due to technical errors or to the need for adjusted dose because of poor economic status of patients (drugs paid for by patients).	Correct drug administration. Drugs paid for by Tanzanian government

Inadequate chemotherapy preparation (no personal protection, absence of laminar flow hood, inadequate dose preparations and wrong modality of drug dilution, i.e., half dose sometimes administered, needles touched by operators). Unclean surfaces before and after chemotherapy preparation. Same syringe used for multiple preparations. No cups and dresses were available	Carbon filtered masks now used for preparation of chemotherapy. Laminar flow hood acquired. Full chemotherapy doses prepared. Drugs diluted using glucose solution when necessary. Surfaces cleaned with sodium hypochlorite is before and after chemotherapy preparation. Single syringe used for each preparation. Presence of cups and dresses.

Drugs prepared and administered in the same room.	Drug preparation occurs in a separate room.

Unskilled operators.	Operators trained in procedures to have a correct management of the therapy and patients. Implementation of hand washing procedures. Pharmacists and technicians now present in BMC Medical Oncology Unit.

Lack of knowledge about therapeutic schedules relating to different cancer types.	Therapeutic schedules relating to different cancer types provided by IRST.

Lack of 250 ml saline solution (solution prepared from bottle of 500 mL saline solution by naked-eye estimation.	250 mL saline solution now available.

Lack of electronic database with patient information (also for toxicity).	Database set up with data inserted on blood tests and clinical pathological characteristics of patients.


***Table 1*** shows the most important issues regarding operator safety and drug appropriateness which had substantially changed by the medical educational program [7]. The data were collected by IRST personnel who, as part of the team of rotating volunteers, made several visits to BMC during the course of the project.

The training courses have made Tanzanian healthcare workers more aware of the risks associated with the handling of anticancer drugs and have increased their understanding of the importance of using personal protective equipment and of recording adverse events and non-conformities. This has led to a lower incidence of adverse events because they have been reported in medical records (e.g., extravasation). A further important issue that has not been neglected is the importance of adherence to international guidelines for cancer therapies to ensure treatment standardization.

## Conclusions

**Figure 3:**
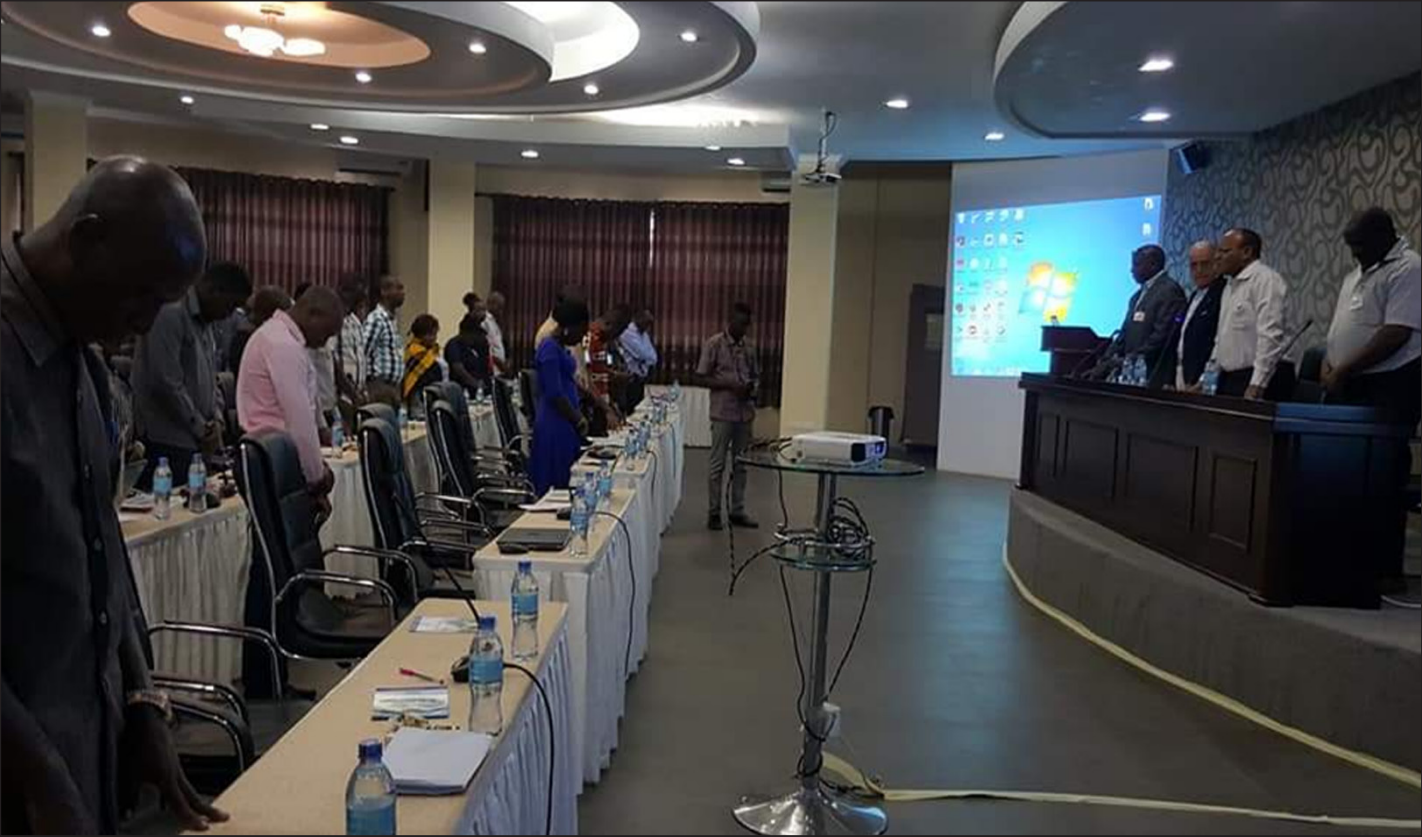
International Conference ‘Global Cancer Control in the 21st Century: a Multidisciplinary Approach’ (Mwanza, 2018).

In conclusion, the training of professionals at BMC is an ongoing process guaranteed by the continuing medical education program established as part of the Mwanza Cancer Project. Since the project began, three international cancer conferences (Infection and Cancer 2012; AORTIC, East African Regional Meeting 2015; Global Cancer Control in the 21^st^ Century: a Multidisciplinary Approach, 2018 (***Figure 3***) and two courses (Theoretical and Practical Course in Oncology 2014 and 2016) have been held at BMC as well as several training stages for physicians, nurses and pharmacists have been organised in Italy. Medical education could be further improved by carrying out collaborative studies and sharing information [6,10,11,12]. The creation of a tutoring system and the setting up of quality control procedures are also essential [7,11,12].

One of the main problems faced by hospitals in Italy is that of maintaining the already high-quality standards in patient care and operator safety. Conversely, such acceptable standards have still not been achieved in Tanzania, highlighting the urgent need to increase the number of cancer hospitals with skilled personnel.

Another critical issue is the availability of drugs to guarantee the continuity of treatment. In general, chemotherapy and radiotherapy (cobalt therapy) are available. Since 2013, trastuzumab has been the only monoclonal antibody accessible for use as targeted therapy, while immunotherapies are still not available. However, the drugs provided by the government are not always obtainable in a sufficient quantity. Furthermore, their cost is covered by the government because the majority of Tanzanian patients do not have sufficient funds to pay for treatments.

The Mwanza Cancer Project has exerted a positive influence on the government’s decisions relating to the reimbursement policy for cancer drugs. The key to this change lies in the creation of a dedicated cancer centre at BMC with diagnosis and treatment services, an infrastructure for drug delivery, oncology-trained personnel, and a system for continuing professional health education. These are prerequisites for ensuring that universal accessibility of medicines may have a significant impact on the disease. The lack of trained personnel is a particularly important barrier to remove in order to expand cancer care services in developing countries. In terms of educational programs and technical support, as a result of the contribution of the Associazione Tison at BMC, Tanzanian operators are now able to teach the acquired processes to local personnel, representing an important step towards achieving that goal.

We plan to carry out a follow-up study to determine whether the improvements made in clinical practice, specifically when it comes to patient management and chemotherapy preparation, have positively impacted the survival and quality of life of Tanzanian cancer patients attending BMC, and also whether, and in what way, the training process has continued independently without our support.
